# Beliefs about illness: comparing foreign- and native-born persons with type 2 diabetes living in Sweden in a cross-sectional survey

**DOI:** 10.1017/S1463423623000269

**Published:** 2023-05-24

**Authors:** Emina Hadziabdic, Katarina Hjelm

**Affiliations:** 1 Department of Health and Caring Sciences, Faculty of Health and Life Sciences, Linnaeus University, Sweden; 2 Department of Public Health and Caring Sciences, Uppsala University, Sweden

**Keywords:** beliefs about illness, diabetes mellitus, migrants, nursing

## Abstract

**Aim:**

Based on previous qualitative studies, it was hypothesised that dissimilarities in beliefs about illness, which influence healthcare-seeking behaviour, exist between foreign- and native-born persons diagnosed with type 2 diabetes living in Sweden (in the following termed ‘Swedish-born’).

**Background:**

Beliefs about illness are individual, culturally related, based on knowledge, and guide health-related behaviour, and thus have an impact on health. The question is whether beliefs differ between foreign- and native-born persons diagnosed with type 2 diabetes. No previous comparative studies have been found on this. Based on previous qualitative studies, it was hypothesised that dissimilarities in beliefs about illness, which influence healthcare-seeking behaviour, exist between foreign- and native-born (Swedish) persons diagnosed with type 2 diabetes living in Sweden.

**Methods:**

Cross-sectional survey, 138 participants, comprising 69 foreign- and 69 Swedish-born persons aged 33–90 vs 48–91 years. Data were analysed with descriptive and analytic statistics.

**Findings:**

Beliefs about illness differed between foreign- and Swedish-born persons concerning causes of diabetes and healthcare-seeking behaviour. Foreign-born persons more often than Swedish-born persons reported uncertainty or lack of knowledge about whether heredity (67% vs 90%, *P* = 0.002) and pancreatic disease (40% vs 62%, *P* = 0.037) could cause diabetes. To a higher extent than Swedish-born persons, they reported that emotional stress and anxiety could cause the disease. Furthermore, they claimed they had sought care due to diabetes during the last 6 months to a higher extent than Swedish-born persons (30% vs 4%, *P* = 0.000).

The findings confirmed that dissimilarities in beliefs about illness, including the causes of diabetes and healthcare-seeking behaviour, exist between foreign- and Swedish-born persons with type 2 diabetes.

## Introduction

Beliefs about illness are individual, culturally related, based on knowledge, and guide health-related behaviour, including self-care and care-seeking, and thus have an impact on health (Hjelm *et al.*, 1999; Hjelm *et al.*, 2003; Hjelm *et al.*, 2005; Hjelm and Bard, 2013). Health-related behaviour is essential for illness control and prevention of complications, in this case diabetes mellitus. Type 2 diabetes is an increasing public health concern among migrants (International Diabetes Federation, 2021). Thus, the question is whether beliefs about illness, including causes of diabetes and healthcare-seeking behaviour, differ between foreign-born persons (who have moved to another country voluntarily or who have been forced to flee as refugees) and Swedish-born persons diagnosed with type 2 diabetes in Sweden. No previous comparative studies have been found on this.

Theoretically, explanations of illness guide strategies for self-care actions and management of disease (Helman, 2007). When ill, a person seeks explanations of the causes of the disease and starts an inner dialogue on how to act. Illness can be perceived as produced by factors related either to the individual, nature, social relations, and/or the supernatural world. The cause/s can be seen as either possible to influence (internal) or outside one’s own control (external), as a result of factors such as luck, chance, fate or the will of God; this is termed ‘internal vs external locus of control’. Those having an internal locus of control and who feel they have control over their health are more likely to carry out health-related behaviours (Rotter, 1966). Also, perceived threat, the seriousness of and susceptibility to a disease, which are included in the health belief model (Rosenstock *et al.*, 1988), exert an influence. The explanation/s and the health beliefs then guide the person to seek advice and care either from family, friends or relatives in the popular sector, health professionals in legally sanctioned institutions in the professional sector, and/or from folk healers in the folk sector (Kleinman, 1980). Where to turn depends on the healthcare system in the society, the availability of resources, individual preferences and experiences, and cultural values/traditions. Regardless of origin, the care-seeking process seems to start with consultation of families, friends, and/or relatives (the popular sector) (Kleinman, 1980; Helman, 2007). In the next step, people in Western societies are more likely to consult healthcare professionals, while folk healers are consulted only in the last instance (Kleinman, 1980; Helman, 2007), but in non-Western countries care seeking is highly dependent on individual socio-demographic characteristics as educated persons are more likely to consult biomedical professionals first before using folk healers (Sato, 2012a; 2012b). Thus, individual beliefs about illness have an impact on health, self-care practices, the type of healthcare sought, and the type of care that is given (Glanz *et al.*, 2015), and it is important to assess individual beliefs in order to provide holistic, person-centred, and integrative care considering both lay and professional beliefs in order to achieve the main goal in nursing: good health or prevention of illness (McFarland and Wehbe-Alamah, 2019).

The single previous comparative study on illness beliefs in persons with diabetes mellitus (with few exceptions type 1) revealed that Europeans cited more causes of diabetes mellitus compared with North Africans, who cited either stress or fate but never heredity or dietary habits food and drink transgression (Dechamp-Le-Roux *et al.*, 1990). Another comparative study seemed similar but focussed on the relationship between illness and treatment perceptions with adherence to diabetes self-care in Arabic-speaking immigrants and Caucasian English-speaking patients (Alzubaidi *et al.*, 2015). Caucasian English-speakers reported a better overall understanding of diabetes and stronger beliefs in their personal ability to control diabetes than Arabic-speaking immigrants, who were significantly less adherent to self-management. Thus, biomedical explanations and folk beliefs are mixed (Kleinman 1980; Helman 2007).

Previous qualitative explorative studies have shown dissimilarities in beliefs about health and illness in persons of different foreign origin compared to Swedish-born people diagnosed with diabetes mellitus (Hjelm *et al.*, 1999, 2003, 2005, Hjelm and Bard, 2013), thus influencing health-related behaviour and care-seeking. The studies have indicated lower knowledge, confirmed in a survey (Pettersson *et al.*, 2019), and risk awareness concerning the perceived seriousness of the disease and its complications in foreign-born than in Swedish-born persons. Foreign-born persons often discussed the presence of post-traumatic disorders (PTSD), the influence of migration experiences, often traumatic, and being an immigrant and experiencing adaptational problems due to language barriers, cultural distance etc. as the cause of diabetes, and many also had a fatalistic view of the disease. It related the causes to factors outside their own control, e.g. fate or supernatural factors such as the will of Allah. This contrasted with Swedish-born persons, who described living a healthy and regular life and related causes to lifestyle and genetics. Use of complementary alternative medicine differed between migrant groups; persons from Latin America described using different types of herbs to a high extent (Hjelm and Bard 2013), while persons from the former Yugoslavia used herbal teas as a complement to prescribed medication (Hjelm *et al.*, 1999, 2003, 2005). In contrast, Middle Eastern-born persons mostly denied the use of complementary alternative medicine (Hjelm *et al.*, 2003, 2005). Different self-care behaviours–active, passive, and information-seeking–were found in Swedish-born persons, former Yugoslavians, Middle Easterners, and Latin Americans. Middle Eastern-born persons also had a lower threshold for seeking care, while persons from the former Yugoslavia described a low inclination for self-monitoring of blood glucose and preventive foot care and often relied on healthcare staff for help. Swedish-born persons often suspected they had contracted diabetes mellitus and thus sought help from healthcare centres in the professional sector while foreign-born persons more often had been admitted to the hospital for other reasons, e.g. infections, fainting etc., and the diagnosis was a secondary finding.

It was thus hypothesised that dissimilarities in beliefs about illness, which influence healthcare-seeking behaviour, exist between foreign-and native-born persons diagnosed with type 2 diabetes living in Sweden (in the following termed ‘Swedish-born’), and the aim of this study was to test this.

More specifically, dissimilarities in beliefs about the causes of diabetes, type of care sought, and care contacts during the last 6 months were studied.

## Methods

### Design

A cross-sectional survey using structured questions was used to compare beliefs about causes of diabetes and healthcare-seeking behaviour between foreign-and Swedish-born persons diagnosed with type 2 diabetes living in Sweden in order to test the hypothesis by examining the relationship between the variables (Creswell, 2014).

### Procedure and participants

Inclusion criteria to participate in the study were persons aged ≥18 years and with a duration of diabetes ≥1 year. The study excluded persons that, in medical records, had identified psychiatric diagnoses (ICD F 00- F29/F60-F 99) because cognitive deficiency might influence the results.

Permission to implement the study was received from the manager of the primary healthcare centre in an immigrant-dense area. After approval, the diabetes specialist nurses in a primary healthcare centre identified individuals who met the inclusion criteria from digital records. A prepaid envelope was sent to identified individuals (*N* = 379). This contained information about the study, the investigator’s contact details, and a request to participate in the study. The information was translated by authorised translators into the most common foreign languages (Arabic and Bosnian/Croatian/Serbian) among foreign-born in Sweden (Statistiska centralbyrån, 2020) and was included in the letter to identify individuals with this background. Individuals who did not respond received two reminders that were sent at intervals of three weeks.

In total, 69 foreign-born persons (response rate 28.5%) were willing to participate in the study (242 persons did not answer, 52 persons did not want to participate in the study, and 14 envelopes were returned because of a wrong postal address). A similar procedure was performed to recruit Swedish-born persons and to match them by gender and diagnosis of type 2 diabetes (ICD E 11) with the foreign-born group.

### Data collection

Data were collected between September 2014 and March 2016 through structured interviews. The interview guide was developed based on previous qualitative studies of individual beliefs about illness (Hjelm *et al.*, 1999, 2003, 2005, 2013) and a review of the literature.

The survey included four measurements of beliefs about illness: beliefs about causes of diabetes (12 items), type of care sought in the first instance (five items), type of care sought in the second instance (five items), and care contacts during the last 6 months (three items). Participants replied to statements in the survey concerning beliefs about the causes of diabetes by giving a response on an ordinal four-point scale: “yes”, “maybe”, “no”, or “do not know”. When analysing the data, the responses “maybe” and “do not know” were combined. Concerning the answer about the type of care sought, the participants were requested to choose between five statements: “healthcare staff at the primary healthcare centre”, “healthcare staff at the hospital”, “someone in my family”, “persons outside the healthcare service who are alternative medicine practitioners”, and “other”. Participants replied to statements in the survey by giving a response on an ordinal three-point scale, “yes”, “maybe”, and “no” to the questions “I have sought care due to the diabetes during the last six months”, “I make regular visits for routine monitoring of the disease”, and “I know when I need to seek care outside the routine visits”.

A registered nurse performed the structured interviews in a secluded location at the primary healthcare center, and in the presence of a professional authorised interpreter when the participants so desired (*n* = 40). During the interviews, the interpreter interpreted what was said literally, using the first person (I-form), was neutral and maintained confidentiality (Kammarkollegiet, 2019).

The interview guide was pilot-tested on 10 foreign-born persons. The pilot interviews were of good quality, and thus all the interviews were included in the study.

### Data analysis

The four measurements of beliefs about illness were analysed with descriptive statistics, and the analyses were performed with the help of SPSS version 26 (SPSS Inc, Chicago, IL, USA). Comparisons between the groups, foreign- and Swedish-born persons, were made in terms of frequencies and percentages and by tests of statistical significance using a chi-squared test and Monte Carlo-based *P*-values significant 2-sided, with *P* < 0.05 considered statistically significant. Furthermore, the influence of education, gender, and ethnic origin (born in Sweden, born in Europe, or born outside Europe) was tested. In the next stage, logistic regression analysis was carried out with the calculation of odds ratio (OR, 95% CI) to assess the statistical significance of the association between dependent variables – influence of heredity as a cause, emotional stress and anxiety as a cause, and pancreatic disease as a cause – and the independent variables that were tested, demographical variables such as educational level (high [university] and low [no education/primary/secondary school]), gender (female and male) and in terms of being foreign-born (born in a European country or born in a non-European country) or native-born (born in Sweden), as significant differences had been found in these.

## Findings

The study included 69 foreign-born person, of whom 37 (54%) were men and 32 (46%) were women, all originating mainly from countries in the former Yugoslavia and the Middle East, and 69 native-born (Swedish-born) persons, of whom 37 (54%) were men and 32 (46%) were women (see Table 1). Foreign-born persons were mostly refugees, and they had shorter duration of type 2 diabetes and poorer glycaemic control than Swedish-born persons.

**Table 1.** Socio-demographic background data of Swedish-and foreign-born persons diagnosed with type 2 diabetes living in Sweden

Variable	Swedish-born	Foreign-born
Country (n)		
Sweden	69	
Outside Europe		38
Syria		14
Iraq		12
Chile		5
Lebanon		3
Sri Lanka		1
Burma		1
Burundi		1
Somalia		1
Europe		31
Bosnia		9
Turkey		8
Poland		4
Finland		4
Kosovo		3
Italy		1
Gender (n (%))		
Male	37 (54)	37 (54)
Female	32(46)	32 (46)
Age (n (%))		
18–64 yrs	25 (36)	44 (64)
>65 years	44 (64)	25 (36)
Educational level (n (%))		
No education	0	10 (15)
Primary school (<9 years)	25 (36)	27 (39)
Secondary school	24 (35)	17 (25)
University ≤ 2 years	7 (10)	9 (13)
University > 2 years	13 (19)	6 (9)
Employment status (n (%))		
Employed	20 (29)	14 (20)
Unemployed	2 (3)	12 (17)
Student	0	2 (3)
Sick leave	1 (1)	11 (16)
Early pension	10 (15)	12 (17)
Retired	39 (52)	18 (26)
Duration of diabetes in years (n (%))		
<10	28 (40)	39 (58)
≥10	41 (60)	30 (42)
Diabetes treatment (n (%))		
Diet		
Oral agents	9 (13)	10 (15)
Insulin	33 (48)	38 (55)
Combination of oral	16 (23)	8 (12)
Agents and insulin	11 (16)	13 (19)
Glycemic control (n (%))		
< 52 (mmol/mol) Good	26 (38)	35 (51)
52–70 (mmol/mol) Acceptable	40 (58)	21 (30)
>70 (mmol/mol) Poor	3 (4)	13 (19)
Cause of migration (n (%))		
Refugee		50 (73)
Employment		7 (10)
Relative		12 (17)
Years of residence in Sweden, mean (SD)		25 (17)

### Beliefs about illness

The majority of respondents, regardless of origin, had not suspected they had diabetes mellitus until they were diagnosed (data not shown: 77% vs 67%, N = 51 vs 45, *P* = 0.395).

Concerning beliefs about the causes of diabetes, there were no differences with the exception of the influence of heredity, emotional stress and anxiety, and pancreatic disease. Foreign-born persons reported to a higher degree that emotional stress and anxiety could cause diabetes (87% vs 53%, *P* = 0.000), and they were unsure or did not know whether heredity (67% vs 90%, *P* = 0.002) and pancreatic disease could cause diabetes, while Swedish-born persons stated these as causes of the disease (62%, *P* = 0.037, see Table 2).

**Table 2.** Beliefs about causes of diabetes among foreign- and Swedish-born persons diagnosed with type 2 diabetes living in Sweden

Beliefs about causes of diabetes		Foreign-born n (%)	Swedish-born n (%)	Total N (%)	*P*-Value
Heredity	Yes	43 (67)	61 (90)	104 (80)	0.002
	No	11 (17)	1 (1)	12 (9)	
	Maybe/don’t know	10 (16)	6 (9)	16 (12)	
Emotional stress, anxiety	Yes	56 (87)	36 (53)	92 (70)	0.000
	No	5 (8)	12 (18)	17 (13)	
	Maybe/don’t know	3 (5)	20 (30)	23 (17)	
Infections	Yes	17 (30)	23 (34)	40 (31)	0.461
	No	19 (31)	14 (21)	33 (26)	
	Maybe/don’t know	25 (41)	30 (45)	55 (43)	
Medicine	Yes	6 (10)	8 (12)	14 (11)	0.841
	No	16 (26)	18 (28)	34 (27)	
	Maybe/don’t know	40 (64)	39 (60)	79 (62)	
Pancreatic disease	Yes	25 (40)	42 (62)	67 (51)	0.037
	No	7 (11)	3 (4)	10 (8)	
	Maybe/don’t know	30 (48)	23 (34)	53 (41)	
Wrong diet	Yes	50 (77)	60 (88)	110 (82)	0.104
	No	8 (12)	2 (3)	10 (7)	
	Maybe/don’t know	7 (11)	6 (9)	13 (10)	
Inactivity	Yes	53 (83)	61 (90)	114 (86)	0.511
	No	5 (8)	3 (4)	8 (6)	
	Maybe/don’t know	6 (9)	4 (6)	10 (8)	
Overweight	Yes	55 (89)	63 (96)	118 (92)	0.361
	No	2 (3)	1 (1)	3 (2)	
	Maybe/don’t know	5 (8)	2 (3)	7 (6)	
Fate	Yes	16 (26)	13 (20)	29 (23)	0.388
	No	35 (57)	37 (56)	72 (57)	
	Maybe/don’t know	10 (16)	16 (24)	26 (20)	
Punishment by God	Yes	3 (5)	0	3 (2)	0.170
	No	54 (87)	62 (95)	116 (91)	
	Maybe/don’t know	5 (8)	3 (5)	8 (6)	
Punishment by the evil eye	Yes	0	0	0	0.535
	No	57 (92)	62 (94)	119 (93)	
	Maybe/don’t know	5 (8)	4 (6)	9 (7)	

When studying the influence of gender, educational level, and ethnic origin, it was shown that being male, having a low level of education, or being of European origin had a negative influence on beliefs about the causes of diabetes (*P* = 0.009, *P* = 0. 026, *P* = 0.10 see Table 3). When adjusting for these factors in logistic regression analysis (see Table 4), being foreign-born and low educational level were still related factors. Believing that heredity-caused diabetes was associated with being foreign-born, irrespective of originating from a European (OR 0.11 (0.02–0.49)) or a non-European (OR 0.15 (0.03–0.63)) country. However, the influence of emotional stress and anxiety as a cause of illness was related to being born in a non-European country (OR 0.15 (0.03–0.63)), as well as to high educational level (OR 3.33 (1.17–9.85)). Considering pancreatic disease as a cause for illness was related to high educational level (OR 3.06 (1.27–7.39)).

**Table 3.** Beliefs about causes of diabetes influenced by gender, educational level, and country of birth among foreign-born and Swedish-born persons diagnosed with type 2 diabetes living in Sweden

		Male				Educational level low				Country of birth				
Beliefs about causes of diabetes		Foreign-born n (%)	Swedish-born n (%)	Total N (%)	***P*** **-Value**	Foreign-born n (%)	Swedish-born n (%)	Total N (%)	***P*** **-Value**	Swedish-born n (%)	Born in Europe n (%)	Born outside Europe n (%)	Total N (%)	***P*** **-value**
Heredity	Yes	23 (64)	33 (92)	56 (78)	0.009	35 (71)	43 (90)	78 (80)	0.026	61 (54)	17 (22)	26 (28)	104 (79)	0.005
	No	6 (17)	0 (0)	6 (8)		9 (18)	1 (2)	10 (10)		1 (1)	5 (18)	6 (17)	12 (9)	
	Maybe/don’t know	7 (19)	3 (8)	10 (14)		5 (10)	4 (8)	9 (9)		6 (9)	6 (21)	4 (11)	16 (12)	
Emotional stress and anxiety	Yes	30 (83)	16 (44)	46 (64)	0.001	43 (88)	20 (42)	63 (65)	0.000	36 (53)	23 (82)	33 (92)	92 (70)	0.000
	No	4 (11)	7 (19)	11 (15)		5 (10)	12 (25)	17 (17)		12 (18)	3 (11)	2 (6)	17 (13)	
	Maybe/don’t know	2 (5)	13 (36)	15 (21)		1 (2)	16 (33)	17 (18)		20 (29)	2 (7)	1 (3)	23 (17)	
Pancreatic disease	Yes	11 (32)	22(61)	33 (47)	0.039	17 (35)	26 (54)	43 (45)	0.051	42 (62)	10 (38)	15 (42)		0.003
	No	6 (18)	2 (6)	8 (11)		6 (13)	3 (6)	9 (9)		3 (4)	0 (0)	7 (19)	10 (8)	
	Maybe/don’t know	17 (50)	12 (33)	29 (41)		25 (52)	19 (40)	44 (46)		23 (34)	16 (61)	14 (40)	53 (41)	

**Table 4.** Multifactorial influence on beliefs about causes of diabetes in foreign-and native-born (Swedish) persons diagnosed with type 2 diabetes living in Sweden. Results from multiple logistic regression analysis with significant influence shown for independent variables

Dependent variable	Independent variables	***P*** **-value**	Odds Ratio (95% CI)
Beliefs about causes of diabetes			
1) Heredity	Low educational level	0.737	0.81 (0.24–2.69)
	Gender (male)	0.841	0.89 (0.32–2.52)
	Country of birth:		
	Born in a European country	0.003	0.11 (0.02–0.49)
	Born in a non-European country	0.009	0.15 (0.03–0.63)
2) Emotional stress and anxiety	Low educational level	0.024	3.33 (1.17–9.85)
	Gender (male)	0.113	1.99 (0.85–4.65)
	Country of birth:		
	Born in a European country	0.007	4.33 (1.49–12.55)
	Born in a non-European country	0.009	0.15 (0.03–0.63)
3) Pancreatic disease	Low educational level	0.013	3.06 (1.27–7.39)
	Gender (male)	0.215	1.59 (0.76–3.34)
	Country of birth:		
	Born in a European country	0.055	0.39 (0.15–1.01)
	Born in a non-European country	0.069	0.45 (0.19–1.06)

Multiple regression analysis was performed in SPSS. Variables with *P* < 0.1 in bivariate analysis were chosen as covariates and entered as categorical variables. Educational level was dichotomised as high (university) and low (no education/primary/secondary school) level education. Gender was dichotomised as female and male. Country of birth was dichotomised as being foreign-born (born in a European country or born in a non-European country) or native-born (born in Sweden). These dependent variables were dichotomised into yes/maybe and no/don’t know.

The majority of the respondents, irrespective of origin, stated individual lifestyle factors such as inactivity and wrong diet as the main causes of diabetes (approximately 70%–96%, see Table 2) rather than the influence of external and supernatural factors such as fate, punishment by God or the evil eye, which were not seen, with few exceptions (*n* = 5), as causing diabetes. However, there were some foreign-born persons who believed that punishment by God can cause diabetes, and a few persons (*n* = 5), irrespective of origin, expressed doubts about the influence of the evil eye (approximately 6%–8%). Approximately 40%–60% of all the respondents expressed doubts about the influence of infectious diseases and medications as causes of diabetes.

### Beliefs about healthcare-seeking

When considering healthcare-seeking behaviour, no dissimilarities were found between foreign-and Swedish-born persons (see Table 5). The majority of respondents (89% vs 94%) reported they turned to the professional healthcare sector and sought help, both in the first and second instance, from staff in the healthcare centre, and a few turned to family and friends in the popular sector. In the second instance, about one-tenth turned to the hospital for help (12% vs 14%). However, compared to Swedish-born persons, foreign-born persons more often reported that they had sought care due to their diabetic disease during the last 6 months (30% vs 4%, *P* = 0.000, see Table 6). Foreign-born persons had a shorter duration of type 2 diabetes (58% vs 40%, *P* = 0.041), poorer glycated haemoglobin (HbA1c 19% vs 4%, *P* = 0.001), were treated with oral agents (55% vs 48%, *P* = 0.143), and more often reported complications related to the eyes (14% vs 0%, *P* = 0.001) than Swedish-born persons.

**Table 5.** Type of care sought during the last 6 months

	Foreign-born n (%)	Swedish-born n (%)	Total N (%)	***P*** **-Value**
When I have problems related to my diabetes, I first turn to:				
Healthcare staff at healthcare centre	57 (89)	64 (94)	121 (91)	0.138
Healthcare staff at hospital	3 (4)	0	3 (2)	
Someone in my family	4 (6)	2 (3)	6 (5)	
Other	1 (1)	2 (3)	3 (2)	
When I have problems related to my diabetes, I turn in the second instance to:				
Healthcare staff at healthcare centre	47 (84)	36 (73)	83 (79)	0.088
Healthcare staff at hospital	7 (12)	7 (14)	14 (13)	
Someone in my family	2 (4)	2 (4)	4 (4)	
Other	0 (0)	4 (8)	4 (4)	

**Table 6.** Care contacts during the last 6 months

		Foreign-born n (%)	Swedish-born n (%)	Total N (%)	***P*** **-Value**
I have sought care due to the diabetes during the last 6 months	Yes	20 (30)	3 (4)	23 (17)	0.000
	No	46 (69)	65 (94)	111 (82)	
	Maybe/don’t know	1(1)	1 (2)	2 (1)	
I make regular visits for routine monitoring of the disease	Yes	67 (100)	68 (100)	136 (100)	
	No	0	0	0	
	Maybe/don’t know	0	0	0	
I know when I need to seek care outside the routine visits	Yes	63 (94)	62 (94)	125 (94)	0.766
	No	2 (3)	3 (5)	5 (4)	
	Maybe/don’t know	2 (3)	1 (1)	3 (2)	

## Discussion

This study is the first to compare beliefs about illness in foreign- and native-born (here Swedish-born) persons, diagnosed with type 2 diabetes. The findings supported the hypothesis generated in previous qualitative studies (Hjelm and Bard, 2013; Hjelm *et al.*, 1999, 2003, 2005) that beliefs about illness differ regarding causes of diabetes and healthcare-seeking behaviour between foreign- and Swedish-born persons. Compared to Swedish-born persons, foreign-born persons more often reported that they were unsure or did not know whether heredity and pancreatic disease could cause diabetes and stated to a higher extent than Swedish-born persons that emotional stress and anxiety were the cause. It was also found that foreign-born persons reported, to a higher degree than the Swedish-born, that they had sought care due to diabetes mellitus during the last 6 months. The beliefs about heredity, pancreatic disease, and emotional stress and anxiety were also influenced by male gender, being low-educated, and being of European origin. Being foreign-born and having a low educational level were still related factors when these factors were adjusted for in binary logistic regression analysis. In this study, foreign-born persons, particularly those of European origin, had a lower educational level than others, which might explain these results.

A difference was found between the studied groups in that the foreign-born persons, to a higher degree than Swedish-born persons, said that they were unsure or did not know whether heredity and pancreatic disease caused diabetes. They also indicated to a higher extent than Swedish-born persons that emotional stress and anxiety could cause diabetes. This finding supports previous qualitative studies (Hjelm *et al.*, 1999; 2003; 2005; Hjelm and Bard, 2013) and in part a previous comparative study investigating Europeans and North Africans mainly diagnosed with type 1 diabetes (Dechamp-Le-Roux *et al.*, 1990) which found that stress or fate could cause the illness but not heredity. Another study (Alzubaidi *et al.*, 2015), which focussed on adherence to diabetes self-care in Arabic-speaking immigrants and Caucasian English-speaking patients, found that biomedical explanations and folk beliefs were mixed. The influence of socio-cultural context on illness beliefs and diabetes self-management in British South Asians (Patel *et al.*, 2015) and beliefs about the causes of type 2 diabetes and the effectiveness of prevention and treatment strategies among Hispanics of Mexican origin in the United States (Coronado *et al.*, 2004) have been explored. British South Asians commonly stated that genetics, diet, and stress were causes of diabetes mellitus and had fatalistic attitudes to diabetes. Further, diabetes self-management was usually a family practice, and alternative food “therapies”, often recommended by social networks, were used (Patel *et al.*, 2015). Many Hispanics believed having a family history of diabetes, eating a diet high in fat or sugar, and minimal engagement in exercise to be the causes of diabetes (Coronado *et al.*, 2004). Emotions such as fear, intense anger or sadness, and depression were also perceived as hastening diabetes onset. The findings in this study could be explained by beliefs about illness (Helman, 2007), level of knowledge about diabetes (Pettersson *et al.*, 2019), and migratory experiences (Berry and Hou, 2021). Individual beliefs about illness are based on knowledge, are culturally determined, are passed on through language and are learned by socialisation in contact with others in the family, other groups, and organisations in the society (Glanz *et al.*, 2015). Furthermore, the influence of migratory experiences related to social and economic inequalities, PTSD, and knowledge about the body and treatment affect the level of self-efficacy and thus explain dissimilarities in beliefs about illness (Hjelm *et al.*, 2003) between the studied groups. These dissimilarities in beliefs about illness are important for self-care practice and care-seeking behaviour; therefore, it is essential to design diabetes healthcare, based on individual beliefs about illness and considering migratory experiences, socio-economic, cultural, and environmental conditions and knowledge about the body and treatment, in partnership between the individual and healthcare staff in order to prevent illness and to achieve the goal of health (McFarland and Wehbe-Alamah, 2019).

Foreign-born persons reported to a higher degree than Swedish-born that they had sought care due to diabetes mellitus during the last 6 months. The foreign-born persons in this study had a shorter duration of type 2 diabetes and had poorer glycaemic control than Swedish-born persons, requiring more visits to healthcare. Other possible explanations for this result could be that: (i) the foreign-born persons had been recently diagnosed and needed more support and information; (ii) they might have had a different type of diabetes, possibly more severe, as has been found among persons from the Middle East (Glans *et al.*, 2008); (iii) they had a more information-seeking behaviour, previously found among persons from the Middle East (Hjelm *et al.*, 2003, 2005); (iv) they might have limited or lacking social networks to turn to for advice and that they come from cultures where people are more passive and rely on help from staff (Hjelm *et al.*, 1999; Hjelm *et al.*, 2003) and finally, (v) an indication of insufficient diabetes education, which may be neither individually nor culturally adapted (Hadziabdic *et al.*, 2020). Diabetes care in Sweden is recommended and based on the national guidelines for diabetes care (Socialstyrelsen, 2018), which means that the majority of patients diagnosed with type 2 diabetes are managed in primary healthcare centres mostly by a general practitioner and a diabetes specialist nurse working in mini-teams. The aim is to achieve good results for the patient, and patients must take great responsibility for their self-care, based on individual beliefs, which is an important factor in patient treatment adherence (Hjelm *et al.*, 1999, 2003, 2005; Hjelm and Bard, 2013). In this study, beliefs about illness were mainly related to factors in the individual, combined with factors related to the nature and social relations in the social sphere in both foreign-and Swedish-born persons. This finding is in contrast to the explanatory model (Helman, 2007), which describes that persons from non-Western countries emphasise the social or supernatural spheres while westerners more often focus on individual factors (above all lifestyle) and factors related to the nature. In the self-care context, individuals may use remedies or therapies from a single care system, i.e. biomedical or traditional/complementary therapies, or blend medicines from different sources for curative purposes based on different factors such as personal health beliefs (Gyasi *et al.*, 2016), time and cost of treatment (Chipwaza *et al.*, 2014; Amegbor, 2017b) and age, gender, relationship status, economic status, and proximity to healthcare services (Amegbor, 2017a). Therefore, it is important not to take anything for granted but instead to search for individual beliefs about illness and tailor the diabetes education model accordingly. For example, focus group discussions, led by a diabetes specialist nurse in collaboration with a multi-professional team, can be used to identify individual beliefs and knowledge as a starting point for further education (Hadziabdic *et al.*, 2020). This would improve knowledge about type 2 diabetes among migrants, and thus increase self-care behaviour and improve health (Hadziabdic *et al.*, 2020) based on holistic, person-centred, and integrative care (McFarland and Wehbe-Alamah, 2019). This is in order to improve knowledge about type 2 diabetes among migrants, and thus increase self-care behaviour and improve health (Hadziabdic *et al.*, 2020) based on holistic, person-centred, and integrative care (McFarland and Wehbe-Alamah, 2019).

### Study limitations

Developing a new, structured questionnaire may be seen as a limitation in this investigation. However, the questionnaire was based on previous qualitative studies of individual beliefs about illness (Hjelm *et al.*, 1999, 2003, 2005; Hjelm and Bard, 2013) and a review of the literature. The interview guide used for the structured interviews was pilot-tested on foreign-born persons for accuracy in the interpretations, and for the understanding of the content, and was interpreted literally by a professional interpreter. Furthermore, various measures were taken to increase survey participation, for example information was sent to the participants about the study, translated by authorised translators into the most common foreign languages (Arabic and Bosnian/Croatian/Serbian) among foreign-born persons in Sweden, and two reminders were sent (Ahlmark *et al.*, 2015). However, the study population did not differ from the non-respondents concerning gender, age, or country of birth. The study results are strengthened by the findings from previous qualitative studies of individual beliefs about illness (Hjelm *et al.*, 1999, 2003, 2005; Hjelm and Bard, 2013) and contribute to a deeper understanding of the importance of assessing individual beliefs about illness, on which to base individual care planning.

## Conclusions

The findings of this study support the hypothesis that dissimilarities in beliefs about illness exist and influence healthcare-seeking behaviour between foreign- and native-born persons living in Sweden diagnosed with type 2 diabetes. Dissimilarities were found in beliefs concerning the influence of heredity and pancreatic disease as causes of diabetes. Foreign-born persons, more often than Swedish-born persons, said that they were unsure or did not know whether heredity and pancreatic disease could cause diabetes. Foreign-born persons also stated to a higher extent than Swedish-born persons that emotional stress and anxiety could cause diabetes. Finally, foreign-born persons reported to a higher degree than Swedish-born that they had sought care due to diabetes mellitus during the last 6 months. Confirmed dissimilarities illustrate the impact on awareness of risk and level of self-care practice. It is important that healthcare services develop models for diabetes education and healthcare that are based on individual beliefs about illness in order to provide holistic, person-centred, and integrative healthcare to maintain health and prevent ill health and the development of diabetes-related complications.

## Data Availability

In order to protect the integrity, anonymity, and confidentiality of the respondents, data will not be shared.
